# Special Issue: Evidence-Based Diagnosis and Management of Facial Nerve Disorders

**DOI:** 10.3390/diagnostics13193056

**Published:** 2023-09-26

**Authors:** Gerd Fabian Volk, Caroline Cora Kraus, Steffen U. Eisenhardt, Shai Rozen

**Affiliations:** 1 Department of Otorhinolaryngology, Jena University Hospital, Am Klinikum 1, 07747 Jena, Germany; caroline.cora.kraus@uni-jena.de; 2 Facial-Nerve-Center Jena, Jena University Hospital, Am Klinikum 1, 07747 Jena, Germany; 3 Center of Rare Diseases Jena, Jena University Hospital, Am Klinikum 1, 07747 Jena, Germany; 4 Department of Plastic and Hand Surgery, Medical Center-University of Freiburg, Faculty of Medicine, University of Freiburg, 79106 Freiburg, Germany; 5 University of Texas Southwestern Medical Center, Dallas, TX 75390, USA

Although there has been a rapid increase in the number of new publications and studies in relation to the diagnostics, impacts and rehabilitation methods of facial nerve disorders, a general structure in evidence-based medicine is still difficult to establish. With this Special Issue on the topic of “Evidence Based Diagnosis and Management of Facial Nerve Disorders”, we brought together experts from various fields, including speech pathology, plastic surgery, otolaryngology, neurology, and physical therapy, to present the latest developments in relation to facial nerve disorders and to show a great variety of topics to our audience/readership. 

There are a few case review studies on this fascinating condition, for example a retrospective examination from 1987 to 2018 on different variants of the treatment for facial nerve schwannoma and changes in facial nerve function afterwards [1], as well as a rare case of facial nerve palsy caused by “osteoradionecrosis of the temporal bone” [2], which is a “[…] rare, delayed complication after radiotherapy for head and neck cancer” [2]. 

Furthermore, the advance of different diagnostic methods and assessment is worthy of observation. The article by A. Kehrer et al., for example, attempts to “[…] outline the key steps in a [high-resolution ultrasound] examination and extract an optimized workflow schema” [3] in patients with post-palsy synkinesis, while other articles focus on electrodiagnostic methods with the aim of creating a more objective evaluation of nerve injuries [4], including the attempt to “[…] develop a standardized protocol for a reliable surface EMG examination of all nine ear muscles in twelve healthy participants” [5] which was then applied “[…] in seven patients with unilateral postparalytic facial synkinesis” [5]. Following that, we can also provide information about more recently developed methods, like the possibility of facial nerve palsy diagnostics via “[…] the use of regional information” [6] from photographs. 

The impacts of facial nerve disorders on daily life, such as communication and also emotion recognition caused by different underlying conditions, are extremely relevant as well, and are intensively discussed in two articles which devote themselves to the subject of emotion recognition. The first one shows the testing of “[…] emotion recognition […] in patients with central facial paresis after stroke” [7] in relation to a healthy control group, while the other one focuses on “[…] the effects of post-paralytic facial synkinesis on facial emotion recognition” [8]. 

Another aspect with utmost importance is the possibility of rehabilitation, which is outlined here in a conservative method described by C. E. Boschetti et al. in the form of a statistic comparison of patients participating or not participating in Kabat physical rehabilitation with “temporary facial nerve palsy after parotid tumor surgery” [9], while the article by H. Abing et al. evaluates an operative method by observing “[…] the time course of clinical and electromyographical (EMG) reinnervation after the reanimation of the smile using a gracilis muscle transplant which is reinnervated with the masseteric nerve” [10]. 

In conclusion, we can see that the topic of facial nerve palsy offers a vast and dynamic spectrum of developments in the fields of causation, diagnostics, and therapy/rehabilitation. We hope that the research presented in this Special Issue shows the importance of a multidisciplinary approach and will not only help to enhance our knowledge of this condition, but also ultimately improve patient outcomes and their quality of life.
